# Exercise in type 2 diabetes: to resist or to endure?

**DOI:** 10.1007/s00125-012-2513-5

**Published:** 2012-03-06

**Authors:** M. Roden

**Affiliations:** 1 Institute for Clinical Diabetology, German Diabetes Center, Leibniz Center for Diabetes Research, Heinrich-Heine University, c/o Auf’m Hennekamp 65, 40225 Düsseldorf, Germany; 2 Department of Metabolic Diseases, Heinrich-Heine University Düsseldorf, Düsseldorf, Germany

**Keywords:** Exercise, Glycaemic control, Physical training, Type 2 diabetes mellitus

## Abstract

There is now evidence that a single bout of endurance (aerobic) or resistance exercise reduces 24 h post-exercise subcutaneous glucose profiles to the same extent in insulin-resistant humans with or without type 2 diabetes. However, it remains to be determined which group would benefit most from specific exercise protocols, particularly with regard to long-term glycaemic control. Acute aerobic exercise first accelerates translocation of myocellular glucose transporters via AMP-activated protein kinase, calcium release and mitogen-activated protein kinase, but also improves insulin-dependent glucose transport/phosphorylation via distal components of insulin signalling (phosphoinositide-dependent kinase 1, TBC1 domain family, members 1 and 4, Rac1, protein kinase C). Post-exercise effects involve peroxisome-proliferator activated receptor-γ coactivator 1α and lead to ATP synthesis, which may be modulated by variants in genes such as *NDUFB6*. While mechanisms of acute resistance-type exercise are less clear, chronic resistance training activates the mammalian target of rapamycin/serine kinase 6 pathway, ultimately increasing protein synthesis and muscle mass. Over the long term, adherence to rather than differences in metabolic variables between specific modes of regular exercise might ultimately determine their efficacy. Taken together, studies are now needed to address the variability of individual responses to long-term resistance and endurance training in real life.

Several randomised controlled trials have demonstrated that moderate changes in lifestyle including both dietary behaviour and physical activity clearly decrease the risk of progression to diabetes in humans with glucose intolerance. Although interventions to increase physical activity raised self-reported physical activity, equivalent to 150–210 min/week of walking, only a few trials reported successful outcomes at more than 12 months [1]. There was also no clear relationship between intervention intensity and physical activity outcomes. While the American College of Sports Medicine (ACSM) and the American Diabetes Association (ADA) jointly recommend that at least 150 min/week of moderate/vigorous physical activity should be undertaken to prevent or delay the development of type 2 diabetes, the recommendations are less firm for physical activity in patients with overt type 2 diabetes [2]. The strength of evidence for a benefit in type 2 diabetes was rated only moderate for intensity, frequency and mode of exercise training.

There are two modes of exercise: endurance and resistance training. Endurance training, generally defined as aerobic exercising involving several muscle groups that aims to improve cardiorespiratory performance, includes activities such as running, biking or swimming. Resistance or strength training forces contraction of defined muscle groups against a defined elastic or hydraulic resistance. While endurance training is considered the first choice for patients with overt type 2 diabetes, the ACSM/ADA statement also recommended that ‘in addition to aerobic training, persons with type 2 diabetes should undertake moderate to vigorous resistance training at least 2–3 days/week’ [2]. This is surprising because the statement also admits that ‘acute effects of resistance exercise in type 2 diabetes have not been reported’ and that ‘more studies are needed to determine whether total caloric expenditure, exercise duration, or exercise mode is responsible’.

In this issue of *Diabetologia*, van Dijk and colleagues [3] report the results of a study that aimed to examine the acute effects of resistance exercise compared with endurance exercise in three different states of insulin resistance and glycaemic impairment. They designed a randomised crossover study with three interventions: a single bout of endurance exercise, a single bout of resistance exercise and a control period. Each study period lasted 3 days and continuous subcutaneous glucose monitoring was performed for 24 h after each intervention. During the experimental periods the participants were free living except for the intervention and a standardised diet. The authors examined three different groups, each comprising 15 middle-aged overweight to obese males: impaired glucose tolerance, type 2 diabetes on oral glucose-lowering agents and type 2 diabetes on insulin. This approach should help to address the following questions:

1. Is acute resistance exercising equal to acute endurance exercising in terms of improving blood glucose levels?
2. Is there a difference in the onset and duration of any beneficial effect between these modes of exercising?
3. Do such effects differ between individuals at risk and those with type 2 diabetes? And, in particular,
4. Does resistance or endurance exercising bear a greater risk of hypoglycaemia in humans with type 2 diabetes on oral glucose-lowering drugs or on insulin?

## Endurance and resistance training equally reduce 24 h glucose post exercising

Overall, glucose concentrations were 7–12% lower in all groups for 24 h after exercising, without significant differences between the modes of exercising. Interestingly, glucose values exceeded 10 mmol/l for an average of about 2 h in glucose-intolerant persons and for more than 8 h in patients with type 2 diabetes. This is in line with observations that even intravenous insulin infusion requires high insulin doses and tight manual control of infusions to restore normoglycaemia in type 2 diabetes [4] and that subcutaneous insulin infusion pumps fail to completely normalise postprandial hyperglycaemia in type 1 diabetes [5]. Nevertheless, both modes of exercise similarly reduced the duration of excess glucose levels by more than 30% in patients with type 2 diabetes, with endurance-type training being gradually more efficient over the course of the first 6 h post exercise.

The duration of sensor glucose levels below 3.9 mmol/l was also not significantly different between control and exercise periods. Of note, the average duration of ‘hypoglycaemia’ (defined as blood glucose concentration <3.9 mmol/l) was generally prolonged in insulin-treated patients (30–39 min) and tended to be longer after endurance-type exercise (24 min) compared with resistance-type exercise (12 min) and control intervention (8 min) in patients on oral agents. In particular, between 6 and 12 h after endurance-type exercising, the duration of low glucose concentrations, albeit still short, was longer in insulin-treated patients.

## Continuous subcutaneous glucose monitoring

While these data suggest an overall comparable efficacy of one bout of either endurance or resistance exercise on glycaemic profiles, they also point to differences in the time course of glucose lowering. For detailed analysis, one should consider the method employed to monitor glucose concentrations. Although the authors converted the data obtained from the continuous glucose monitoring sensor into ‘blood glucose concentrations’ using the person’s self-monitored capillary glucose data, these values reflect subcutaneous interstitial rather than blood glucose concentrations. Although the sensor device used by van Dijk et al. [3] seems to provide a tight linear correlation between blood and sensor glucose values in patients with type 1 diabetes, sensor data underestimate glycaemia during high intensity exercise and overestimate glycaemia during low intensity exercise [6]. In addition, sensor data exhibit a delayed response compared with blood glucose during exercise recovery. Moreover, only 87% of the sensors from another provider gave adequate signals for 24 h in exercising adolescents with type 1 diabetes [7]. Data on the accuracy of sensors in exercising patients with type 2 diabetes are still rare and the challenge to correctly assess hypoglycaemia has been mentioned by the senior author of the present paper before [8]. Of note, older patients with insulin resistance or long-standing type 2 diabetes may also have altered adipose tissue blood flow, which might differently affect blood and interstitial glucose levels [9]. In any case, these limitations affect the absolute numbers rather than the overall conclusions of this paper.

## Acute exercise

**Fig. 1:**
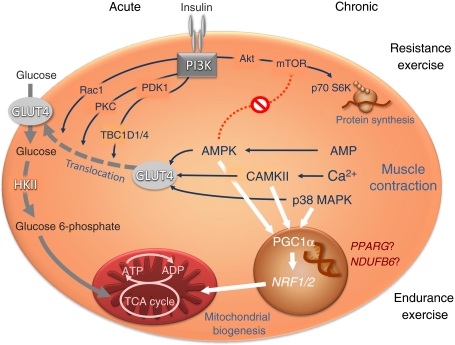
Mechanisms of exercise-stimulated glucose uptake in skeletal muscle. Upon acute endurance-type exercise, muscular contraction increases AMP and AMP-activated protein kinase (AMPK), calcium release from endoplasmic reticulum and Ca^2+^/calmodulin-dependent protein kinase II (CAMKII) as well as mitogen-activated protein kinase (MAPK). This will accelerate translocation of GLUT4 and thereby facilitate insulin-independent glucose transport. Post-exercise effects involve activation of peroxisome-proliferator activated receptor-γ coactivator 1α (PGC1α), which by stimulating expression of nuclear respiratory factors (*NRF1*, *NRF2*) will increase ATP synthesis and, later, mitochondrial biogenesis. In addition, exercising improves insulin-dependent glucose transport and phosphorylation by hexokinase II (HKII), which includes more distal components of insulin signalling such as phosphoinositide-dependent kinase 1 (PDK1), TBC1 domain family, members 1 and 4 (TBC1D1/4), Rac1 and protein kinase C (PKC) isoforms. The effects of training on mitochondrial function and glucose metabolism may be modulated by variants in genes such as *PPARG* or *NDUFB6*. While the cellular mechanisms of acute resistance-type exercise are less clear, chronic resistance training activates the mammalian target of rapamycin (mTOR)/serine kinase 6 (S6K) pathway, ultimately leading to protein synthesis and increased muscle mass. TCA, tricarboxylic acid

Other issues need to be considered when assessing the efficacy of resistance vs endurance training on glycaemic control in dysglycaemic states. In their study, van Dijk et al. [3] compared the glucose responses to only a single bout of each exercise protocol, which may be subject to considerable day-to-day variability. Also, the short lag time of 4 days between the different interventions does not completely rule out carry-over effects of previous exercise on insulin sensitivity, which could last up to 6 days or more [10]. After acute endurance-type exercise, several cellular pathways (Fig. 1), mainly AMP-activated protein kinase, calcium signalling and p38 mitogen-activated protein kinase, stimulate myocellular glucose transport [11] and may also increase flux through ATP synthase in vivo, even in relatives of patients with type 2 diabetes [12]. In addition, muscle insulin sensitivity improves in insulin-resistant humans following one bout of endurance exercising, as demonstrated by increased insulin-stimulated glucose transport/phosphorylation [13]. While no changes were found at the level of the insulin receptor, its substrates or phosphoinositide 3-kinase, and more distal components of insulin signalling, such as protein kinase C isoforms, Rac1 and TBC1 domain family, member 4 may be more active [14] (Fig. 1). Resistance exercise training activates the mammalian target of rapamycin/serine kinase 6 pathway, thereby increasing protein synthesis and muscle mass [15]. Now, van Dijk et al. [3] provide evidence for a glucose-lowering effect, obviously occurring independently of muscle mass. This is in line with a previous report demonstrating that even a single session of resistance exercise can improve fasting glucose and insulin sensitivity as calculated by HOMA [16]. This paper further addressed the question of whether exercise intensity affects glycaemia and suggested a dose–response relationship between programme variables of volume and intensity and 24 h post-exercise insulin sensitivity. Thus, the conclusions of the present paper are likely valid only for the intensity of the exercise prescription chosen.

Would individuals experience hyperglycaemia owing to the greater counter-regulatory hormone release associated with intense resistance exercise, or would those with impaired counter-regulation owing to long-standing well-controlled diabetes be at greater risk of hypoglycemia? More data on metabolic and hormonal responses to acute exercising including plasma profiles of NEFA, catecholamines, glucagon and growth hormone are needed to answer this question. Thus, the results apply only to middle-aged to elderly patients with insulin resistance and type 2 diabetes, but not to younger patients and those with type 1 diabetes with a lesser degree of insulin resistance and differences in hypoglycaemia counter-regulation.

## Chronic exercise

The study by van Dijk et al. [3] was not designed to address the clinically relevant question of whether endurance- or resistance-type exercise training should be the first choice for recommendations on chronic physical activity in type 2 diabetes. In this context, a recent meta-analysis showed that structured aerobic, resistance or combined exercise training results in a decline in HbA_1c_ ranging from 0.51 to 0.73 percentage points in patients with type 2 diabetes, with an exercise duration of >150 min/week being more efficient than a duration of ≤150 min/week (reduction in HbA_1c_ of 0.89 vs 0.36 percentage points) [17]. While the analysis suggests equal clinical efficacy of the various types of exercise in type 2 diabetes, the different numbers, sizes and designs of the studies included do not allow a final conclusion to be drawn.

It has been assumed that chronic endurance training might act primarily by improving insulin resistance, while resistance training would augment the capacity for glucose uptake by increasing skeletal muscle mass. In addition, chronic exercise training can increase levels of PGC1α and, in turn, expression of genes encoding nuclear respiration factors to stimulate mitochondrial biogenesis and function (Fig. 1), both of which are frequently impaired in insulin-resistant and diabetic states [18]. Endurance exercise training performed three times weekly markedly improved whole body insulin sensitivity and normalised muscular glucose transport/phosphorylation within 6 weeks in first-degree relatives of patients with type 2 diabetes [13]. Combined endurance and resistance exercise for 12 weeks also normalised muscular mitochondrial density and capacity in individuals with type 2 diabetes, although insulin sensitivity remained reduced [19]. On the other hand, both resistance and exercise training twice weekly improved ATP synthase flux in vivo but failed to increase insulin sensitivity in first-degree relatives [20]. Interestingly, the response of mitochondrial function was modified in part by a single nucleotide polymorphism (SNP) in the *NDUFB6* gene, which encodes a subunit of complex 1 of the respiratory chain (Fig. 1) [20]. A SNP in the *PPARG* gene may further modulate the response of glucose metabolism to regular endurance exercise [21]. These recent reports underline the important roles of intensity and frequency of exercise training regardless of the mode of exercise and also point to individual, inherited predisposition defining the success of interventions aiming to increase physical activity.

Taken together, the findings of van Dijk et al [3] provide evidence for the comparable efficacy of one bout of a specific protocol of endurance or resistance exercise on post-exercise glycaemic control in insulin-resistant humans with or without type 2 diabetes. But it remains to be determined who will benefit most from specific exercise protocols with regard to long-term glycaemic control and insulin sensitivity. Not only cellular mechanisms, but also long-term acceptability by patients, can differ between endurance and resistance training. Consequently, long-term adherence to exercise advice rather than specific modes of exercise might ultimately determine efficacy to improve glycaemia and, importantly, morbidity and mortality. As a result, studies are now needed to determine whether we should resist or endure when exercising in the long run in real life.

## Abbreviation

ACSM: American College of Sports Medicine
